# The Comprehensive Evidence on the Effectiveness and Experiences of Mentoring Programmes for Managers in Healthcare: A Mixed‐Methods Systematic Review

**DOI:** 10.1155/jonm/9921900

**Published:** 2026-08-26

**Authors:** Mari Elmi, Suvi Kuha, Eevi Karsikas, Outi Kanste

**Affiliations:** ^1^ Research Unit of Health Sciences and Technology, University of Oulu, Oulu, Finland, oulu.fi; ^2^ The Finnish Centre for Evidence-Based Health Care: A Joanna Briggs Institute Centre of Excellence, Helsinki, Finland; ^3^ Medical Research Center Oulu, Oulu University Hospital, University of Oulu, Oulu, Finland, oulu.fi

## Abstract

**Aim:**

The aim of this mixed‐methods systematic review is to synthesise and integrate the evidence on the effectiveness of mentoring programmes in terms of managers’ leadership competencies and managers’ experiences of mentoring programmes in healthcare.

**Background:**

Mentoring has a significant impact on leadership in healthcare. It offers benefits for mentors, mentees and organisations by increasing job satisfaction, retention and cost savings, as well as improving patient outcomes. Its importance for new or prospective managers and managerial students is well recognised within training and academia. The existing evidence on mentoring programmes for managers in healthcare is scattered; therefore, comprehensive data on mentoring programmes for managers, encompassing both effectiveness and experiences, are needed.

**Methods:**

A mixed‐methods systematic review was conducted in accordance with the Joanna Briggs Institute methodology for mixed‐methods systematic reviews. A systematic search of the CINAHL (EBSCO), Scopus, Medic, Web of Science, Medline (Ovid), ProQuest and MedNar databases was carried out in November 2023 and updated in August 2025. Literature published in English, Finnish and Swedish was searched, with no restrictions on the year of publication. A narrative summary of quantitative data and a thematic synthesis of qualitative data were used to integrate the findings, following the convergent segregated approach.

**Results:**

A total of ten studies were included. The integration of quantitative and qualitative evidence yielded three comprehensive themes: managerial skills and knowledge reinforcing managers’ professional growth, increased soft skills improving managers’ personal growth and diligent arrangements ensuring managers’ growth.

**Conclusions:**

Mentoring programmes were shown to have beneficial effects on the development of different leadership competencies. Managers experienced positive improvements in their personal and professional growth. This synthesis underlines the importance of mentoring programmes for managers and provides ideas for future development, including advocating for flexible and diverse models with tailored support and time allocation.

## 1. Introduction

Healthcare is currently facing multiple increasingly complex challenges. Therefore, there is a need for dedicated, skilled and motivated healthcare workers [1], with an emphasis on competent management workforces to drive change [2]. A growing number of studies [3] and interventions are being conducted to enhance the leadership competencies of healthcare managers [4], as their roles continue to expand and require skills such as business management, leadership and self‐development [5]. Leadership competencies can be defined as a combination of leadership knowledge, skills, attitudes and behaviours that are necessary for individuals in managerial roles [2].

Managers have many complex and evolving roles in constantly changing healthcare systems [6, 7]. Expanding scientific knowledge [8], a lack of time management, the unique complexity demands [9], rapid technological developments [10] and the lack of alignment between personal values and professional duties require strong competencies and organisational support [11, 12]. The challenges faced by managers may result in feelings of frustration and reduced job satisfaction, ultimately leading to burnout [9] and a decision to leave their position [13]. Hence, there is a need to consider methods to support managers in healthcare organisations [14]. Mentoring has been identified as one method to address this need [4, 15].

The concept of mentoring has existed for many years, but it remains ambiguous [16]. However, it has gained popularity as a formal concept in healthcare education and learning over the last 20 years [17] and is considered an essential step in improving leadership competencies [4]. Mentoring is defined in higher education as a relationship between two people with the specific purpose of one assisting the other to grow and develop and to reinforce success, competence and career development [18]. Based on the concept analysis by Hodgson and Scanlan [19], nurse leadership mentoring is characterised as a relationship between two individuals with differing levels of experience. Mentoring describes a method of employee development in which an experienced mentor provides guidance, encouragement, career advice and support to another person [19]. It is based on mutual respect and common goals involving knowledge sharing and active engagement by both parties [19–21].

Mentoring programmes can be beneficial for both mentees and mentors [22] while also contributing to overall benefits for organisations [23]. Mentoring is associated with increased job satisfaction and retention, cost savings and improved patient outcomes. Organisations have a crucial role in decision‐making when addressing the mentoring needs of their employees [15]. The types of mentoring have expanded as workplace technology and relationships have become more complex. Traditional mentoring involves a senior professional mentoring a junior. As programmes have developed, value has also been found in group [24], reverse [25], remote [26] and e‐mentoring [27]. Mentoring programmes require a clear format and objectives to achieve success [22]. This involves organisational support [28], including dedicating time and resources to mentorship activities and creating an enabling environment for mentees to receive mentoring [22]. In addition to formal mentoring programmes, it has been suggested that informal mentoring in organisations can foster beneficial interactions between potential partners, underlining the mentee’s role as an active entity who sets their own goals and is also accountable for the effectiveness of the mentoring [15, 29].

Based on the points discussed above, mentoring programmes for managers need to be sustained and supported in healthcare organisations. Due to concerns about burnout and turnover of managers, Lyle‐Edrosolo et al. [13] identified major themes for future research. These themes included the improvement of leadership development programmes and the need to evaluate the effectiveness of this approach. A systematic review by Chen et al. [4] highlighted the importance of incorporating qualitative process evaluations alongside studies assessing leadership interventions to gain a deeper understanding of participants’ perceptions of these interventions. This study provides an assessment of the effectiveness of mentoring programmes in terms of improving managers’ leadership competencies in healthcare. In addition, this study provides an overview of managers’ experiences with mentoring programmes, with the intention of understanding their perspectives to gather information that will inform future changes and guide healthcare organisations in developing successful mentoring programmes.

A preliminary search of PROSPERO, Scopus, Medline (Ovid), ProQuest Database and Google Scholar was conducted, and no current or in‐progress systematic reviews on the topic were identified. The benefits of mentoring in academia [30–37] and health research [38, 39] have been well‐documented in recent reviews, which provide support for mentoring as a means to enhance research productivity, career development [32, 33, 36] and leadership competencies [34, 37, 40]. A systematic review by Vlerick et al. [41] found that mentoring programmes had positive outcomes for specialised and advanced practice nurses. Similar findings were reported by Keinänen et al. [42] in their review, which found that mentoring education programmes effectively enhanced mentoring competencies among healthcare professionals. In contrast to previous reviews [30–42], this scoping review has been refined to focus solely on participants holding managerial positions. Regarding the mentoring of managers, a 2013 study by Hodgson and Scanlan [19] considered the concept of mentoring and its relevance to nursing leaders. Although previous studies have reported on the benefits of mentoring in various areas of healthcare, no review has yet been published on the use of mentoring for healthcare managers. This study addresses a knowledge gap concerning both the effectiveness and experiences of mentoring programmes for managers in healthcare.

## 2. Aim and Review Questions

The aim of this mixed‐methods systematic review (MMSR) is to synthesise and integrate the evidence on the effectiveness of mentoring programmes in terms of managers’ leadership competencies and managers’ experiences of mentoring programmes in healthcare. This review addresses two questions:

1. How effective are mentoring programmes in terms of improving healthcare managers’ leadership competencies?
2. What are the experiences of managers of mentoring programmes in healthcare?

## 3. Methods

### 3.1. Study Design

A MMSR was conducted in accordance with the Joanna Briggs Institute (JBI) methodology for MMSRs, following the convergent segregated approach. Due to the complex nature of the review topic and the different types of information, MMSR was selected as the review method. MMSR offers a more comprehensive synthesis of the evidence compared to single‐method reviews. By combining quantitative data on intervention effectiveness and qualitative data reflecting the experiences, MMSRs enhances the applicability and relevance of the synthesised findings, thereby providing more robust and contextually grounded evidence to inform clinical decision‐making, policy development and practice improvement [43]. This review was reported in accordance with the Preferred Reporting Items for Systematic Reviews and Meta‐Analyses (PRISMA) criteria [44]. The protocol was registered with PROSPERO, the International Prospective Register of Systematic Reviews (CRD42023474237).

### 3.2. Inclusion and Exclusion Criteria

The inclusion and exclusion criteria for quantitative and qualitative studies were based on PEO (Participants, Exposure of interest, Outcomes [45]); and PICo (Participants, Phenomena of interest and Context [43]) guidelines. Quantitative studies were eligible for inclusion if they met the following criteria: (1) participants were healthcare managers at any level of management; (2) the exposure of interest was the effectiveness of mentoring programmes in terms of leadership competencies; and (3) the outcomes assessed included dimensions of leadership competency, encompassing knowledge, skills, abilities, attitudes, values, judgement and personal attributes.

Qualitative studies were included if they: (1) involved participants who were healthcare managers at any level of management; (2) explored the phenomena of interest in the experiences, perceptions, attitudes and understandings related to mentoring programmes; and (3) were conducted within the context of healthcare settings and organisations (Table 1).

**TABLE 1:** Inclusion and exclusion criteria for quantitative studies (PEO) and for qualitative studies (PICo).

Inclusion criteria for quantitative studies (PEO)		Exclusion criteria
Participants (P)	Healthcare managers at any level of management	Students and professionals not currently in management roles
Exposure of interest (E)	The effectiveness of mentoring programmes in terms of leadership competencies	No documentation of the effectiveness of mentoring programmes in terms of leadership competencies
Outcomes (O)	Leadership competency outcomes, including outcomes relating to knowledge, skills, abilities, attitudes, values, judgement and personal attributes	No documentation of measurements of leadership competencies, including knowledge, skills, abilities, attitudes, values, judgement and personal attributes

**Inclusion criteria for qualitative studies (PICo)**		**Exclusion criteria**

Participants (P)	Healthcare managers at any level of management	Students and professionals not currently in management roles
Phenomena of interest (I)	Experiences, perspectives, attitudes and understanding of mentoring programmes	No documentation of experiences, perspectives, attitudes or understanding of mentoring programmes
Context (Co)	Healthcare settings and organisations	Nonhealthcare settings and organisations

*Note:* Source: Authors’ own work.

### 3.3. Search Strategy

The search strategy aimed to find both published and unpublished studies. A three‐step search strategy was utilised for this review. First, an initial limited search of PubMed and CINAHL (EBSCO) was conducted, followed by an analysis of the article titles and abstracts, as well as the index terms used to describe the articles. The search strategy, including all identified keywords and index terms, was adapted for each included information source. A second search was conducted in November 2023, and the search was updated in August 2025. Studies published in English, Finnish and Swedish were included with no date limitation. The database search included CINAHL (EBSCO), Scopus, Medic (a Finnish database), Web of Science, Medline (Ovid) and ProQuest Databases. Sources of unpublished studies and grey literature were searched on MedNar. The search strategy was developed in cooperation with an information specialist (Supporting Table 1). Finally, the reference lists of all selected studies were screened for additional relevant studies.

### 3.4. Study Selection

**FIGURE 1:**
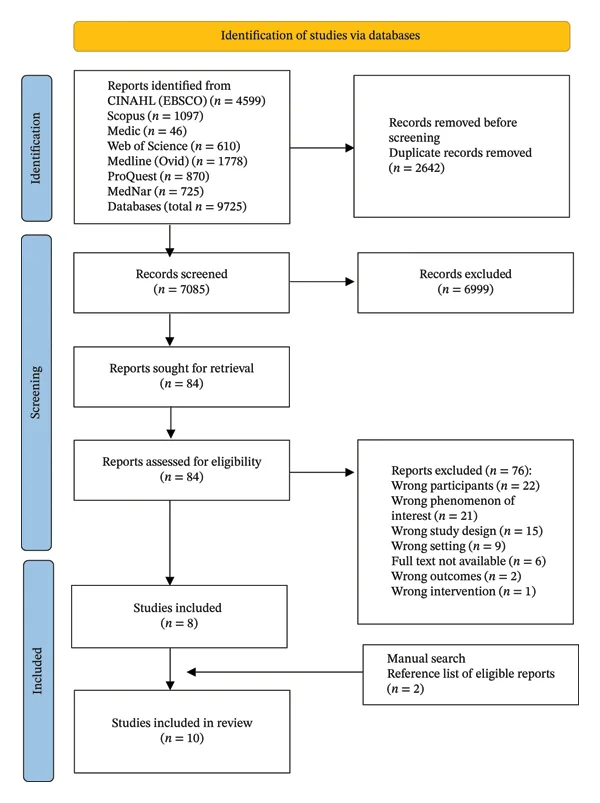
Flowchart of the study selection process (Page et al. [44]).

The database search identified 9725 studies as being potentially relevant to the review. Following the search, all identified citations were collated and uploaded into the Covidence Systematic Review Software tool [46]. After the removal of 2642 duplicates, the titles and abstracts of 7085 studies were screened by four independent reviewers (authors blinded). Any disagreements that arose between the reviewers were resolved through discussion or by a third reviewer. A total of 6999 studies were excluded as irrelevant, and the full texts of 84 studies were retrieved. Of these, eight full‐text studies met the inclusion criteria and were included in the critical appraisal. The reference lists of included articles were searched for additional relevant studies, and two [47, 48] were identified. Therefore, the review included a total of ten studies. The search results and selection process are presented in the PRISMA flow diagram (Figure 1). For more information about the reasons for exclusion can be obtained from the corresponding author.

### 3.5. Quality Assessment

Three independent reviewers critically appraised eligible studies (authors blinded) for methodological quality using JBI standardised critical appraisal instruments [49] for quasi‐experimental studies [50], analytical cross‐sectional studies [45] and qualitative studies [51]. For mixed‐methods studies, the quantitative and qualitative components were appraised separately using the appropriate JBI critical appraisal instruments. Any disagreements that arose between the reviewers were resolved through discussion. All studies were included regardless of their methodological quality.

The included quasi‐experimental studies scored 7 [52, 53] or 8 [47, 48, 54, 55] out of 9, the analytical cross‐sectional studies scored 6 [56] or 8 [57] out of 8, and the qualitative studies scored from 5 to 8 [55, 56, 58, 59] out of 10 (Supporting Table 2). In quasi‐experimental studies, most deficiencies were related to the absence of a control group. In cross‐sectional studies, the most common deficiencies concerned the identification of confounding factors and the articulation of strategies to address these confounders. In qualitative studies, most concerns were related to the congruence between the stated philosophical perspective and the research methodology, the researcher’s cultural or theoretical positioning, and the consideration of the researcher’s influence on the research and vice versa.

### 3.6. Data Extraction and Synthesis

Quantitative and qualitative data were extracted from the included studies by the first author using Microsoft Word (Microsoft Corporation, Redmond, WA) and crosschecked by the other reviewers. The extracted data included author, year of publication, country, study purpose, participants, methodology, type of mentoring and key findings (Supporting Table 3).

This review followed the convergent segregated approach to synthesis and integration in accordance with the JBI methodology for MMSRs. A convergent segregated approach was selected to accommodate the distinct research questions addressed by the quantitative and qualitative evidence. This approach enabled the separate analysis of quantitative and qualitative data, which were subsequently integrated and combined. The synthesis of both data types provides complementary evidence, offering a more comprehensive understanding of the phenomenon of interest [43].

In line with JBI guidelines, the quantitative studies were analysed using narrative synthesis [45], with studies grouped according to reported outcomes to summarise the main findings. Qualitative studies were analysed using thematic analysis [60], with the initial data extracted being coded line by line and organised into descriptive themes (*n* = 39) by similarities. These descriptive themes were then grouped into subthemes (*n* = 13) and analytical themes (*n* = 4). Following the segregated approach, the qualitative and quantitative synthesised findings were juxtaposed and integrated to produce an overall configured analysis [43].

## 4. Findings of the Review

### 4.1. Characteristics of Included Studies

The studies were conducted in the United States (*n* = 6), Canada (*n* = 1), Ethiopia (*n* = 1) and Australia (*n* = 1), with one (*n* = 1) being undertaken in several countries as an international study. The earliest study was published in 2003, while the latest study was published in 2023. All the studies were reported in English. The participants were healthcare executives [57], hospital leaders [53], nurse managers [47, 59] or nurse leaders [48, 52, 54–56, 58] who had been mentored. Among the included studies, six examined mentor–mentee pairs [53–56, 58, 59], and the remainder examined only mentees [47, 52, 57]. The number of participants regarded as mentees ranged from 14 to 131 for each study, totalling 280.

Regarding the types of mentoring programmes, in four studies, the mentoring programmes started with an educational programme and subsequently involved various follow‐up sessions or workshops [47, 52, 55, 58]. Four studies involved traditional mentor–mentee meetings [52, 53, 57, 59], while four other studies examined mentoring programmes that included both online and face‐to‐face meetings [47, 48, 55, 58]. One study focused on a mentoring programme exclusively reliant on online or virtual technology [56]. The mentoring model of the remaining study was not clearly defined [54].

Of the ten identified studies, four were doctoral dissertations, and the remainder were research articles. All the quantitative studies (*n* = 6) used surveys to collect data. The mixed‐methods studies (*n* = 4) employed surveys, focus groups, or interviews for data collection; two of these studies reported quantitative outcomes, and all four reported qualitative outcomes.

### 4.2. Quantitative Evidence

**FIGURE 2:**
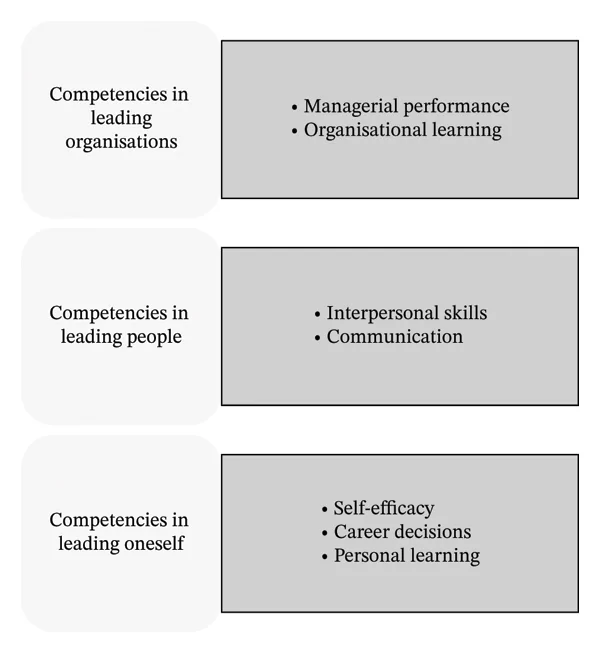
Themes identified in the included quantitative studies on the effectiveness of mentoring programmes on managers’ leadership competencies. *Source:* Authors’ own work.

The narrative synthesis assessed the effectiveness of mentoring programmes in terms of managers’ leadership competencies under three themes: competencies in leading organisations, competencies in leading people and competencies in leading oneself (Figure 2).

#### 4.2.1. Competencies in Leading Organisations

Mentoring programmes were reported to improve managers’ organisational leadership competencies by improving managerial performance [53, 56] and developing organisational learning [55, 56]. Mentoring programmes can be an effective approach to enhance managerial performance by developing human resources and improving hospital operations and nursing management skills [53]. They can influence quality improvements, budgeting and financial management [53], as well as actions to increase leadership activities, thereby contributing to overall leadership development [56]. The studies showed that mentoring developed managers’ organisational learning as it influenced their organisational involvement [56] and fostered a broader understanding of their job and the entire organisation, including its politics and other departments’ functions [55].

#### 4.2.2. Competencies in Leading People

Mentoring programmes were found to influence managers’ people leadership competencies by developing interpersonal skills [53–55] and communication [55]. Mentoring programmes were perceived to have a significant positive impact on participants’ interpersonal skills [54, 55]. The identified studies demonstrated improvements in relationships [53] and advancements in participants’ understanding of how their own department impacts others, as well as their comprehension of the issues and problems faced by other departments [55]. It was reported that mentoring positively influenced managers’ communication, particularly in terms of listening skills and sensitivity towards others, as well as its effects on learning about others’ perceptions of oneself or one’s job [55].

#### 4.2.3. Competencies in Leading Oneself

The results indicate that mentoring programmes had an impact on managers’ self‐leadership competencies, as they were found to influence self‐efficacy [48, 52, 54], career decisions [47, 48, 55–57] and personal learning [47, 54, 55]. The results of studies examining managers’ self‐efficacy varied. It was shown that mentoring programmes did not predict leadership self‐efficacy with statistical significance [52, 54]. However, one study found a statistically significant increase in self‐efficacy [48]. Overall, the general trend across the studies showed that mentoring programmes can enhance overall self‐efficacy among managers [48, 52, 54].

Mentoring programmes were found to influence managers’ career decisions [47, 48, 55–57] by supporting career development [47] and informing them of new ideas related to their job performance [55]. Mentoring programmes were shown to increase managers’ perceptions of their readiness for promotion [48] and help them achieve leadership development goals [56]. Furthermore, some evidence suggests that executives mentored earlier in their careers are more likely to ascend to senior executive roles and subsequently serve as mentors to others [57]. The impact on personal learning was described as significant [47, 54, 55], with mentoring resulting in an increase in leadership competency, transformational leadership behaviours, job satisfaction [47], new personal skills [55] and feelings of self‐competence [54]. However, one study found that the level of confidence in management skills did not increase significantly [53].

### 4.3. Qualitative Evidence

The thematic analysis identified four analytical themes that covered managers’ experiences of mentoring programmes in healthcare: mentoring strengthening managers’ growth; mentoring enhancing managers’ introspection; developing a rewarding mentoring relationship; and benefiting from a successfully implemented mentoring programme (Table 2).

**TABLE 2:** An overview of descriptive themes, subthemes and analytical themes related to managers’ experiences of mentoring programmes.

Descriptive themes (*n* = 39)	Subthemes (*n* = 13)	Analytical themes (*n* = 4)
Facilitating growth in conflict management Approaching difficult, confrontational situations Helping deal with the complex nature of healthcare	Developing skills in managing challenging situations via mentoring	Mentoring strengthens manager’s growth

Building internal and external relationships Improving communication Enhancing networks	Improving skills in interactional communication through mentoring	

Gaining appreciation of the wider issues Developing methods in resource management Improving recognition strategies Building leadership capability	Developing leadership efficacy through mentoring	

Driving engagement among emerging leaders Helping support new nurse leaders	Encouraging new nurse managers by mentoring	

Adopting different management and leadership styles Being aware of leadership activities Gaining insight into skills by clear expectation setting	Improving self‐awareness in leadership through mentoring	Mentoring enhances manager’s introspection

Knowing one’s personal limits Gaining insights from mistakes Talking about shortcomings Being nondefensive Giving opportunities to reflect on personal development	Being open to self‐reflection during mentoring	

Considering the mentor–mentee relationship essential for success Building a good mentor–mentee relationship through engagement Appreciating flexibility in the mentoring relationship	Building the relationship between mentor and mentee	Developing a rewarding mentoring relationship

Gaining trust for crucial conversations Feeling comfortable expressing concerns and fears Having a safe environment for learning Lack of trust	Achieving a confidential atmosphere in mentoring	

Having opportunities to share knowledge with others Sharing ideas to achieve successful outcomes	Enabling knowledge sharing during mentoring	

Receiving advice from the mentor Receiving guidance from the mentor regarding the proper path	Receiving guidance from the mentor during mentoring	

Receiving continued external support from the organiser Receiving good support from mentors Receiving support from the mentoring community	Receiving extensive and varied support	Benefitting from a successfully implemented mentoring programme

Experiencing finding time for mentoring is challenging Appreciating personal timing choices during mentoring	Considering timing in mentoring	

Appreciating individual decisions on conducting mentoring relationships Receiving an appropriate mentoring partner based on shared experience Having a carefully selected mentor partner	Highlighting the selection of mentor and mentee pairs	

*Note:* Source: Authors’ own work.

#### 4.3.1. Mentoring Strengthening Managers’ Growth

The theme of mentoring strengthening managers’ growth comprised four subthemes: developing skills in managing challenging situations through mentoring, improving skills in interactional communication through mentoring, developing leadership efficacy through mentoring, and encouraging new nurse managers through mentoring. Managers felt that mentoring developed their skills in managing challenging situations, as it facilitated growth in conflict management capacities [55, 58]. Mentoring was also described as effective in helping individuals deal with the complex nature of healthcare [58] and in approaching difficult, confrontational situations [55].

It has been shown that mentoring improves skills in interactional communication, including building relationships both internally and externally [58] as well as globally [56], while also enhancing communication skills [55, 58]. Communication skills improved [58] through learning new communication methods and identifying emotions before conveying messages [55]. In addition to relationship building and communication skills, mentoring was mentioned as enabling network enhancement [58, 59] by helping maintain professional networks [59] and improving network knowledge [58].

Managers found that mentoring programmes helped develop their leadership efficacy by providing insight into broader issues [59], developing resource utilisation methods, improving effective recognition strategies [55], and building overall leadership capabilities [56]. The results also indicated that mentoring encourages new nurse managers [56, 58, 59]. This was experienced as a channel to support new nurse managers [58, 59] and drive engagement by activating and contributing to a sense of satisfaction [56].

#### 4.3.2. Mentoring Enhancing Manager’s Introspection

Mentoring enhancing managers’ introspection included two subthemes: improving self‐awareness in leadership through mentoring and being open to self‐reflection during mentoring. The perception of improved self‐awareness in leadership showed that mentoring helped managers explore and implement various management and leadership styles [56] and develop personal awareness of their leadership activities [55, 56]. Especially when clear expectations were set, managers believed they gained good insight into the skills they had achieved [56].

Being open to self‐reflection during mentoring included experiencing one’s personal strengths, weaknesses and limitations [58], as well as having further opportunities to reflect on personal development [59]. Gaining insight from mistakes, maintaining a nondefensive attitude and discussing shortcomings receptively were considered essential aspects of the mentoring experience [58].

#### 4.3.3. Developing a Rewarding Mentoring Relationship

Developing a rewarding mentoring relationship comprised four subthemes: building the relationship between mentor and mentee, achieving a confidential atmosphere during mentoring, facilitating knowledge sharing during mentoring and receiving guidance from the mentor during mentoring. Building the relationship between mentor and mentee was emphasised as key to success [56, 58]. Creating a good mentor–mentee relationship through engagement was experienced as requiring support, connection [55], commitment [59] and development at both the personal and professional levels [58]. Flexibility in the mentoring relationship was emphasised by mentors’ ability to meet managers’ expectations [56], contributing to success [58]. Achieving a common understanding of flexibility within relationships was also considered an essential element of building mentoring relationships [55].

The results show that managers experienced the need to achieve a confidential atmosphere in mentoring [55, 58]. Managers considered that gaining trust and comfort within relationships enabled crucial conversations [58], especially when expressing fears and concerns [55]. A safe environment for learning and growth was also reported to promote managers’ growth and development [58]. Difficulties at the start of a confidential relationship were also experienced as a lack of trust [55].

Enabling knowledge sharing during mentoring consisted of having the opportunity to share knowledge with others [55] and sharing ideas to achieve successful outcomes [56]. The knowledge and experience each participant brought to the mentoring relationship were viewed as a strength [55], and successful outcomes were achieved through the mutual sharing of ideas within relationships [56]. Managers also found that receiving guidance from a mentor was crucial, as mentors were expected to serve as trusted advisors whom managers could seek out for advice [58] while also guiding them on the proper path [56] so that they could become successful leaders [55].

#### 4.3.4. Benefitting From a Successfully Implemented Mentoring Programme

Benefitting from a successfully implemented mentoring programme comprised three subthemes: receiving extensive and varied support, considering timing in mentoring and highlighting the selection of mentor and mentee pairs.

Managers emphasised the importance of receiving support from various sources, including organisers, mentors and other participants, when participating in a mentoring programme [56, 58, 59]. It was reported that ongoing support and educational intervention from the programme throughout the mentoring played an essential role in increasing preparedness [58]. Furthermore, the provision of a highly structured and well‐facilitated programme also involved high expectations of continued extensive support from both the organiser and mentors [59]. A supportive community was also mentioned as an essential source of support [56].

Considering timing was experienced as a crucial factor in mentoring [55, 56, 58, 59]. Challenges finding time to meet [55, 56] and foster mentoring initiatives [58] were mentioned. A desire to choose the timing of mentoring sessions to align with individual schedules and needs was also noted [59]. In addition to timing, the results indicated that careful selection of mentor and mentee pairs is needed in mentoring [55]. The results show that managers highlighted the selection of mentor–mentee pairs. Managers appreciated the ability to choose their own mentors. It was mentioned that matching mentoring partners based on shared experience is better than doing so based on factors such as age or gender [59].

### 4.4. Integration of Quantitative and Qualitative Evidence

The quantitative and qualitative subthemes were synthesised into three comprehensive themes: managerial skills and knowledge reinforcing managers’ professional growth, increased soft skills improving managers’ personal growth, and diligent arrangements ensuring managers’ growth (Table 3).

**TABLE 3:** Integration of quantitative and qualitative evidence into three comprehensive themes.

Quantitative subthemes	Qualitative subthemes	Synthesised comprehensive themes
• Managerial performance • Organisational learning • Career decisions	• Developing skills to manage challenging situations • Developing leadership efficacy • Encouraging new nurse managers	Managerial skills and knowledge reinforcing managers’ professional growth

• Self‐efficacy • Personal learning • Interpersonal skills • Communication	• Improving self‐awareness in leadership through mentoring • Being open to self‐reflection during mentoring • Improving skills in interactional communication through mentoring	Increased soft skills improving managers’ personal growth

	• Building the relationship between mentor and mentee • Achieving a confidential atmosphere in mentoring • Enabling knowledge sharing during mentoring • Receiving guidance from mentor during mentoring • Receiving extensive and varied support • Considering timing in mentoring • Highlighting the selection of mentor and mentee pairs	Diligent arrangements ensuring managers’ growth

*Note:* Source: Authors’ own work.

#### 4.4.1. Managerial Skills and Knowledge Reinforcing Managers’ Professional Growth

Mentoring programmes were shown to reinforce managers’ professional growth by enhancing their managerial skills and knowledge. The results indicate that managers improved their managerial skills in conflict management [55, 58], human resources, hospital operations, nursing management, quality improvement, budgeting and finance management [53]. Managerial skills that were shown to improve included developing new resource utilisation methods and effective recognition strategies [55], coping with the complexities of healthcare [58] and building overall leadership capabilities [56]. It was also reported that mentoring programmes supported managers’ organisational knowledge by fostering a broader understanding of issues [55, 59], including their job, the organisation, its politics and other departments’ functions [55]. Furthermore, the results indicated that mentoring programmes influenced managers’ organisational involvement [56] and career development [47]. Mentoring programmes were reported to improve managers’ readiness for promotion [48, 57], expand their perspectives on job performance [55], drive engagement and help attain leadership development goals [56]. Mentoring programmes were experienced to enable professional growth, particularly among new managers [58, 59].

#### 4.4.2. Increased Soft Skills Improving Managers’ Personal Growth

Mentoring programmes were demonstrated to have an effect on managers’ personal development by enhancing their soft skills. According to the results, mentoring programmes increase managers’ soft skills, particularly in terms of self‐efficacy [48, 52, 54], self‐competence [47, 54], self‐reflection [58, 59] and self‐awareness [55, 56]. The results indicate that improved soft skills help managers implement various leadership skills [47, 55, 56], define personal strengths and weaknesses [58] and reflect on personal development [59]. Mentoring programmes were also determined to enhance managers’ personal growth in soft skills by fostering relationships [53, 56, 58] and networks [58, 59]. The results emphasised that managers improved their interpersonal skills [54, 55] and communication [55, 58], as well as knowledge related to these social interactions [58].

#### 4.4.3. Diligent Arrangements Ensuring Managers’ Growth

The results emphasised that diligent arrangements were essential for ensuring managers’ growth. Diligent arrangements included building mentoring relationships based on support, connection [55], commitment [59], trust, comfort [55, 58], the sharing of knowledge [55, 56] and guidance [55, 56, 58]. The experiences demonstrated that mentoring relationships were crucial for managers’ overall growth and success [55, 56, 58]. Diligent arrangements included managers appreciating the opportunity to select their mentor [59] and having protected time [55, 56, 58, 59] and flexibility for mentoring [55, 56, 58]. When mentoring managers, there was a need for careful selection of mentor–mentee pairs [55]. In addition, the results indicated the importance of extensive programme support [56, 58, 59].

## 5. Discussion

This review compiled a total of ten original studies published between 2003 and 2023, providing more profound and comprehensive information on the effectiveness of mentoring programmes for managers in healthcare and their experiences. The quantitative evidence showed that mentoring programmes had an impact on managers’ competencies in leading organisations, leading people and leading oneself. The qualitative evidence also identified improvements in managers’ growth in all these areas, underlining positive experiences within confidential mentoring relationships and the successful implementation of mentoring programmes. The independent syntheses of quantitative and qualitative evidence largely supported each other. Three comprehensive themes emerged: the managerial skills and knowledge that reinforce managers’ professional growth, the increased soft skills that improve managers’ personal growth, and diligent arrangements that ensure managers’ growth. These themes provided deeper and more nuanced insights into the effectiveness of mentoring programmes in terms of managers’ leadership competencies and their experiences with mentoring programmes in healthcare.

Our integrated results showed that the importance of reinforcing managers’ professional growth in terms of managerial skills and knowledge is a comprehensive theme of mentoring programmes for managers. The narrative synthesis of quantitative evidence was congruent with the synthesised analytical theme of mentoring strengthening managers’ growth. These results indicate that mentoring programmes can improve the overall managerial skills [53, 55, 56, 58] and knowledge [55, 56, 59]. These competencies are needed when developing effective leadership capabilities and conflict management skills. Moreover, our results indicate that managerial skills and knowledge play a vital role in fostering managers’ comprehension of the organisation and encouraging their active involvement within it. The quantitative studies revealed that mentoring influenced managers’ career decisions [47, 48, 55–57] by increasing their readiness for promotion and career development, whereas the qualitative studies did not explore similar career aspects. However, new managers require support and encouragement to advance professionally [58, 59], a point highlighted in the qualitative results. Previous studies have reported similar findings regarding the enhancement of leadership success through mentoring [61, 62], as well as leadership skills and knowledge [34, 37]. Wurmser and Kowalski [63] also noted that mentoring programmes can be an effective mechanism to support leaders in challenging healthcare roles. The role of mentoring in supporting emerging managers is also well recognised in previous studies [64, 65]. Furthermore, our findings align with the recent study by Leonard et al. [66], which found increased interest in future career and leadership positions among student leaders participating in a peer mentoring programme. These aspects should be taken into consideration in succession planning to attract and retain new nurse managers within healthcare organisations.

This review highlighted that mentoring programmes can improve managers’ personal growth by increasing their soft skills. The quantitative results differed regarding the importance of improvements in managers’ self‐competence [47, 54] and self‐efficacy [48, 52, 54]. In support of these findings, the qualitative evidence showed that mentoring programmes encourage managers to engage in self‐reflection [58, 59] and enhance self‐awareness [55, 56]. In addition, these findings highlight the value and significance of mentoring programmes in terms of managers’ interpersonal skills and opportunities for networking. The narrative synthesis of quantitative studies indicates that mentoring programmes can improve interpersonal skills [54, 55]. This is supported by the qualitative findings, which showed that mentoring programmes also improved skills in building relationships [56, 58] and networks [58, 59], as well as advanced communications skills [55, 58]. In accordance with these results, student leaders’ confidence [66, 67] and nurse leaders’ self‐efficacy [68] have been found to improve after mentoring in previous studies. Similar findings concerning enhanced interactions have also been reported in other studies [4, 34, 40, 67], while Rodríguez et al. [69] stated that the development of professional networks is critical for sustained leadership success. Based on our findings, mentoring programmes can be crucial for managers’ longer‐term personal growth, as they lead to the maintenance of these networks and improve the knowledge within them.

Our studies show the need for diligent arrangements to ensure managers’ growth. Managers wanted their mentors to guide and support their development in a safe environment. This review highlights that mentoring relationships are critical to managers’ success, with flexibility [55, 56, 58], mentor–mentee pairing [55], protected time [55, 56, 58, 59] and programme support [55, 56, 58] identified as crucial factors to managers. Support and developmental relationships have also been described as key elements of mentoring in previous studies [18, 21, 24, 67, 70]. Consistent with our results, lack of time has been reported as a common challenge in mentoring across various studies [28, 33, 34, 36, 71–73], as well as the importance of matching mentors with mentees [18, 21, 70]. Additionally, the need for diverse mentoring programmes was recognised in the studies by Abdollahi and Heshmati Nabavi [30] and Chong et al. [27], which examined features of distance mentoring. This underscores the demand for robust assessment tools to guide different mentoring programmes effectively [74]. These factors should also be taken into consideration when implementing mentoring programmes to align the skills and needs of managers better.

## 6. Limitations

This MMSR was conducted according to a carefully followed protocol, with three researchers involved in every phase of the review process. Nevertheless, this review has some limitations. First, it only included studies available in English, Swedish or Finnish, meaning relevant studies in other languages may have been missed. Second, the data extraction and analysis were conducted by a single researcher, increasing the possibility of subjectivity in the analysis. To mitigate this risk, the analysis was discussed and cross‐checked with the other reviewers to enhance credibility. Third, although a library information specialist was consulted during the development of the search strategy, there was a potential for bias in the search strategy, as two of the ten included studies were identified from reference lists. Fourth, the quality of the included studies was evaluated using JBI critical appraisals, and the limited methodological quality of some of the included studies may increase the risk of bias. Regarding the data, statistical pooling was not possible due to the heterogeneity of the studies and findings. Therefore, the findings could only be presented in narrative format.

## 7. Conclusions

This MMSR provided a comprehensive synthesis of the effectiveness of mentoring programmes in terms of managers’ leadership competencies and managers’ experiences of mentoring programmes in healthcare. Mentoring programmes have various beneficial effects on managers, including reinforcing their professional growth by enhancing managerial skills and knowledge, as well as improving their personal development by developing soft skills. Additionally, diligent arrangements are needed for these programmes to ensure managers’ growth. Managers require extensive support and consider timing from mentors and organisations to enact the competencies developed in the mentoring programme.

Future studies should include stronger evidence on the effectiveness of different mentoring programmes, especially those focusing on technology and utilising artificial intelligence for managers, to inform future mentoring models aimed at managers in healthcare. More rigorous experimental research designs are also needed. In addition, qualitative methods are required to gain a deeper insight into participants’ experiences and perceived effects, enabling the adaptation of programmes to meet the needs of managers.

## 8. Implications for Nursing Management

Mentoring programmes should include flexible and diverse models that provide managers with diverse opportunities for mentoring interaction. When considering mentoring programmes to develop managers’ leadership competencies, it is crucial to consider assessment and professional environments for building mentoring relationships with tailored support and time allocation. This review emphasises the importance of providing encouragement and support for managers to achieve the desired outcomes of mentoring programmes and ensure they address the diverse and complex challenges within the healthcare field.

## Author Contributions

Drafting the manuscript: Mari Elmi, Suvi Kuha, Eevi Karsikas, and Outi Kanste. Acquisition of data: Mari Elmi, Suvi Kuha, Eevi Karsikas, and Outi Kanste. Analysis and interpretation of data: Mari Elmi, Suvi Kuha, and Outi Kanste. Writing the manuscript: Mari Elmi. Commenting on the manuscript: Suvi Kuha, Eevi Karsikas, and Outi Kanste.

## Funding

No funding was received for this study.

## Ethics Statement

The study is based on literature and did not require ethical approval.

## Conflicts of Interest

The authors declare no conflicts of interest.

## Supporting Information

Additional supporting information can be found online in the Supporting Information section.

## Supporting information


**Supporting Information** The supporting information includes three files that provide further information about the search strategies, assessment of methodological quality and a list of the selected articles for analysis.

## Data Availability

The data that support the findings of this study are available in the supporting information of this article.
