# Supplementary material for: The Comprehensive Evidence on the Effectiveness and Experiences of Mentoring Programmes for Managers in Healthcare: A Mixed‐Methods Systematic Review
Source: J Nurs Manag. 2026 Aug 26;2026:9921900. doi: 10.1155/jonm/9921900 (PMC13519296; doi:10.1155/jonm/9921900)
Supplement: Supplementary file 1 [file JONM-2026-9921900-s001.docx]

Supplementary Table 1. Databases, search strategy and results by databases.

| Databases | Search strategy | n |
| --- | --- | --- |
| CINAHL (EBSCO) | ( ( ( (MH "Mentorship") OR (MH "Preceptorship") ) AND ( (MH "Leadership") OR (MH "Management+") OR (MH "Leaders+") OR (MH "Administrative Personnel+") ) ) OR ( ( mentor* OR mentee* OR preceptor* ) N5 ( manag* OR leader* OR supervisor* OR administrat* OR executive* OR director* OR charge OR head ) ) ) AND ( (MH "Professional Competence+") OR (MH "Professional Knowledge+") OR (MH "Job Satisfaction+") OR (MH "Attitude of Health Personnel+") OR (competenc* OR knowledge OR skill* OR capability OR qualification OR literacy OR attitude* OR performanc* OR perception* OR experienc* OR view* OR opinion* OR satisf* OR expect* OR attitude* ) ) AND ( nurs* OR "health care" OR healthcare OR hospital* ) NOT ( (MH "Students+") OR student* ) | 3,843 |
| Scopus | ( TITLE-ABS-KEY ( mentor* OR mentee* OR preceptor* ) W/5 TITLE-ABS-KEY ( manag* OR leader* OR supervisor* OR administrat* OR executive* OR director* OR charge OR head ) AND TITLE-ABS-KEY ( competenc* OR knowledge OR skill* OR capability OR qualification OR literacy OR attitude* OR performanc* OR perception* OR experienc* OR view* OR opinion* OR satisf* OR expect* OR attitude* ) AND TITLE-ABS-KEY ( nurs* OR "health care" OR healthcare OR hospital* ) AND NOT TITLE-ABS-KEY ( student* ) ) | 883 |
| Medic | mentor* perehdyt* työnohja* Preceptorship valmen* AND johta* päällik* osastonhoitaj* ylihoitaj* ylilääkär* esimie* esihenkilö* hallin* "Administrative Personnel" Leadership "Organization and Administration" AND kompeten* osaami* taito* kyky* kyvyt* tieto* taitav* taid* asenne* asente* mielipi* näkökulm* kanta* kannat* käsity* kokemu* työtyytyväi* työhyvinvoi* pystyvyy* "Professional Competence" "Job Satisfaction" "Attitude of Health Personnel" | 41 |
| Web of Science | ( mentor* OR mentee* OR preceptor* ) NEAR/5 ( manag* OR leader* OR supervisor* OR administrat* OR executive* OR director* OR charge OR head ) (Topic) and competenc* OR knowledge OR skill* OR capability OR qualification OR literacy OR attitude* OR performanc* OR perception* OR experienc* OR view* OR opinion* OR satisf* OR expect* OR attitude* (Topic) and nurs* OR "health care" OR healthcare OR hospital* (Topic) not student* (Topic) | 479 |
| Medline (Ovid) | (((exp Mentors/ or exp Preceptorship/) and (exp Administrative Personnel/ or exp Leadership/ or exp "Organization and Administration"/)) or ((mentor* or mentee* or preceptor*) adj6 (manag* or leader* or supervisor* or administrat* or executive* or director* or charge or head)).ab,kf,ti.) and (exp Professional Competence/ or exp Job Satisfaction/ or exp "Attitude of Health Personnel"/ or (competenc* or knowledge or skill* or capability or qualification or literacy or attitude* or performanc* or perception* or experienc* or view* or opinion* or satisf* or expect* or attitude*).ab,kf,ti.) and (nurs* or "health care" or healthcare or hospital*).ab,kf,ti. not (exp Students/ or student*).ab,kf,ti. | 1614 |
| ProQuest Databases | noft(( mentor* OR mentee* OR preceptor* ) NEAR/5 ( manag* OR leader* OR supervisor* OR administrat* OR executive* OR director* OR charge OR head ) ) AND noft(competenc* OR knowledge OR skill* OR capability OR qualification OR literacy OR attitude* OR performanc* OR perception* OR experienc* OR view* OR opinion* OR satisf* OR expect* OR attitude*) AND noft(nurs* OR "health care" OR healthcare OR hospital*) NOT noft(student*) | 782 |
| MedNar | ( mentor* OR mentee* OR preceptor* ) AND ( manag* OR leader* OR supervisor* OR administrat* OR executive* OR director* OR charge OR head ) AND ( competenc* OR knowledge OR skill* OR capability OR qualification OR literacy OR attitude* OR performanc* OR perception* OR experienc* OR view* OR opinion* OR satisf* OR expect* OR attitude* ) AND ( nurs* OR "health care" OR healthcare OR hospital* ) NOT student* | 474 |
| Search conducted 6 November 2023. No date limitation applied. | | |

**Source**: Authors’ own work

Supplementary Table 2. Assessment of methodological quality of the included studies.

| **JBI Critical Appraisal Checklist for Qualitative Research** | Hedenstrom et al., 2023 | Phelps, 2005 | Rosser et al., 2020 | Waters et al., 2003 |
| --- | --- | --- | --- | --- |
| 1. Is there congruity between the stated philosophical perspective and the research methodology? | No | Yes | No | No |
| 2. Is there congruity between the research methodology and the research question or objectives? | No | Yes | Yes | Yes |
| 3. Is there congruity between the research methodology and the methods used to collect data? | Yes | Yes | Yes | Yes |
| 4. Is there congruity between the research methodology and the representation and analysis of data? | Yes | Yes | Yes | Yes |
| 5. Is there congruity between the research methodology and the interpretation of results? | Yes | Yes | Yes | No |
| 6. Is there a statement locating the researcher culturally or theoretically? | No | Unclear | No | No |
| 7. Is the influence of the researcher on the research, and vice- versa, addressed? | No | Yes | No | No |
| 8. Are participants, and their voices, adequately represented? | Yes | Yes | Yes | Yes |
| 9. Is the research ethical according to current criteria or, for recent studies, and is there evidence of ethical approval by an appropriate body? | No | No | Yes | No |
| 10. Do the conclusions drawn in the research report flow from the analysis, or interpretation, of the data? | Yes | Yes | Yes | Yes |
| Total score | 5/10(50%) | 8/10(80%) | 7/10(70%) | 6/10(60%) |

| **JBI Critical Appraisal Checklist for Quasi-Experimental Studies** | Blastorah, 2009 | Bradley et al., 2008 | Jean-Luis, 2020 | | McCarthy and Knestrick, 2023 | | Phelps, 2005 | Roth and Whitehead, 2019 |
| --- | --- | --- | --- | --- | --- | --- | --- | --- |
| 1. Is it clear in the study what is the ‘cause’ and what is the ‘effect’ (i.e. there is no confusion about which variable comes first)? | Yes | Yes | Yes | | Yes | | Yes | Yes |
| 2. Were the participants included in any comparisons similar? | Yes | Yes | Yes | | Yes | | Yes | Yes |
| 3. Were the participants included in any comparisons receiving similar treatment/care, other than the exposure or intervention of interest? | Unclear | Yes | Yes | | Yes | | Yes | Yes |
| 4. Was there a control group? | No | No | No | | No | | No | No |
| 5. Were there multiple measurements of the outcome both pre and post the intervention/exposure? | Yes | Yes | Yes | | Yes | | Yes | Yes |
| 6. Was follow up complete and if not, were differences between groups in terms of their follow up adequately described and analyzed? | Yes | Unclear | Yes | | Yes | | Yes | Yes |
| 7. Were the outcomes of participants included in any comparisons measured in the same way? | Yes | Yes | Yes | | Yes | | Yes | Yes |
| 8. Were outcomes measured in a reliable way? | Yes | Yes | Yes | | Yes | | Yes | Yes |
| 9. Was appropriate statistical analysis used? | Yes | Yes | Yes | | Yes | | Yes | Yes |
| Total score | 7/9(78%) | 7/9(78%) | 8/9(89%) | | 8/9 (89%) | | 8/9(89%) | 8/9(89%) |
| **JBI Critical Appraisal Checklist for Analytical Cross-Sectional Studies** | | | | Finley, 2005 | | Rosser et al., 2020 | | |
| 1. Were the criteria for inclusion in the sample clearly defined? | | | | Yes | | Yes | | |
| 2. Were the study subjects and the setting described in detail? | | | | Yes | | Yes | | |
| 3. Was the exposure measured in a valid and reliable way? | | | | Yes | | Yes | | |
| 4. Were objective, standard criteria used for measurement of the condition? | | | | Yes | | Yes | | |
| 5. Were confounding factors identified? | | | | Yes | | No | | |
| 6. Were strategies to deal with confounding factors stated? | | | | Yes | | No | | |
| 7. Were the outcomes measured in a valid and reliable way? | | | | Yes | | Yes | | |
| 8. Was appropriate statistical analysis used? | | | | Yes | | Yes | | |
| Total score | | | | 8/8 (100%) | | 6/8 (75%) | | |

**Source**: Authors’ own work

Supplementary Table 3. Data extraction table for included qualitative, quantitative, and mixed methods studies.

| Author(s), year, and country | Purpose | Participants and context | Methodology (design, data collection, data  analysis) | Type of mentoring | Key findings |
| --- | --- | --- | --- | --- | --- |
| Blastorah, 2009 Canada | To examine the effect of mentoring on nurses’ leadership self-efficacy when provided within the context of a leadership training initiative. | Registered nurses (n=100) who were attending leadership programs, the Dorothy M. Wylie Nursing Leadership Institute and the Health Leaders Institute. The majority of participants had experience in formal leadership positions. Mentored participants (n=61) were included in the main analysis. | A prospective, repeated measures design. Doctoral dissertation. Demographic questionnaire, the mentoring functions questionnaire and the modified LPI-Self questionnaire. Data were collected via mailed survey and analyzed using hierarchical regression analysis in SPSS 15.00. Paired t-tests were conducted on LSE scores. | Comprehensive leadership development intervention that provided leadership training and mentoring. Intervention's structure consisted of a 5-day educational program and a follow-up session for three to four months. The program used developmental strategies includingdidactic presentations, small group work, guided self-reflection, and application of learning to a home-based project. Nearly half of the respondents reported meeting with their mentor at least once per week. | Mentoring did not contribute to the development of leadership self-efficacy in nurses when provided in conjunction with leadership training. Protégé exposure to mentoring functions did not predict leadership self-efficacy. Mentoring was suggested to enhance nurses LSE during leadership training, and to sustain the increase upon return to their home organizations. |
| Bradley *et al.,* 2008,  Ethiopia | To evaluate the impact of a large-scale intervention on several key management indicators through hospital quality improvement efforts. | Hospital leaders and management mentors in government hospitals (n=14) across Ethiopia. Three of the hospitals were located in rural settings, four were located in semi-urban settings and seven were located in Addis Ababa. | Pre– post-descriptive exploratory study. The key management indicator checklist and close ended Likert scale instrument. Data were  analyzed with descriptive statistics and frequency analysis. | A countrywide quality improvement initiative using partnership-mentoring model in which 24 management mentors were placed for 1 year in Ethiopia to work side-by-side with hospital management teams. | The partnership–mentoring model can be an effective approach to management improvement efforts.  Management skills of hospital leaders improved in human resources, hospital operations, nursing management, infection prevention and control, quality improvement, budgeting and finance management domains, although reported confidence in these skills remained largely unchanged. Hospital leaders were able to improve their relationships between medical directors and several key constituent groups. |
| Finlay, 2005, USA | To review the extent to which mentoring is used in the healthcare field as a method to prepare future leaders. | Senior-level healthcare executives (n = 131) from United States | Descriptive study. Doctoral dissertation. The data collected through a survey by mail. Correlation analysis. | Mentoring as a method for preparing future healthcare leaders. | Job tenure, organization tenure, and proximity to retirement were not strongly correlated to levels of support for mentoring.  The study provides some support for the relationship between executives mentored earlier in their career having a great likelihood to advance to senior executive-level positions. Healthcare executives who benefited from a past mentoring relationship will be more likely to serve as mentors. |
| Hedenstrom *et al.,* 2023, USA | To evaluate the effectiveness of a nurse leader mentoring pilot program using Mentoring Up as a theoretical framework. | Mentor (n=15) and mentees (=15) with leadership positions from an acute care hospital in the southeast United States. The mentors had at least 3 years in leadership positions, while the mentees had less than 2 years in the current leadership role. | A mixed-method design. Surveys and focus groups were conducted at mid- and endpoints. Due to a low response rate on a mentoring assessment tool at the end of the study, the qualitative study data were used to report the findings. The constant comparative method was used to code data and identify themes. Data were digitally recorded, transcribed verbatim, and loaded into NVivo 12. | After the 3-hour program kick-off the mentor and mentee met monthly for up to 1 hour at a mutually agreed-upon time over a 6-month period. In addition, midsession focus groups were held. Program consisted of workshops and online sessions. | Mentoring attributes that facilitated a positive mentoring relationship included interpersonal skills and organizational skills. Interpersonal skills included a mentor-mentee relationship that was built upon trust, flexibility, and learning and development; and organizational skills included building relationships both internally and externally. Time was identified as a barrier to mentoring.  Mentoring was described effective in helping deal with the complex nature of health care. Mentees felt mentoring gave them opportunity to grow in conflict management. Many were eager to seek and receive feedback, gain insights from mistakes, receptive to talking about shortcomings, be nondefensive, and know their strengths, weaknesses, and limits.  Educational intervention and support throughout the program contributed to increased preparedness. Mentoring relationships started to develop at both a personal and professional level after the pair got to know and trust one another. Building relationships with other leaders were critical skillsets to harness. |
| Jean-Luis, 2020, USA | To implement a 4-month mentorship program that empowered minority nurse leaders who desire career advancement to executive leadership roles and provide them with realistic tools to manage their professional development. | Ethnically diverse mid-level nurse leaders (n=8) employed by a large, urban academic medical center. | Pre- post study design. Doctoral dissertation.  Leadership Efficacy Questionnaire and the Michigan Organization Assessment Questionnaire. Data were analyzed using descriptive statistics and two paired t-test in SPSS 25 software. | Four group leadership development sessions. Mentors and mentees met at least once every two weeks for individual mentorship. Individual mentorship session were virtually or in person, with at least one live session per month, four months | Perceptions of self-efficacy significantly increased after program participation, (p < .001). Perceptions of readiness for promotion significantly increased after program participation (p < .01). |
| McCarthy and Knestrick, 2023, USA | To discuss the role of mentorship to prepare nurse leaders to serve on boards. | Nurse leaders' mentor and mentee pairs (n=22) from United States. Mentees (n=11) were nurse leaders and mentors (n=11) nurse leaders who had experience in board service. | Pre– post- study design. SHIP-BC survey data and Mentorship Competency Assessment tool. Data were analyzed using descriptive statistics and the Wilcoxon signed rank test. | The 8 weeks evidence-based mentorship program was implemented as a pilot project, where nurse leaders were given the opportunity to build relationships with mentors. | Results showed an increase in self-efficacy and satisfaction with the program among both mentees and mentors. Although the overall change in self-efficacy score measured by the SHIP-BC survey was not statistically significant, the impact of the program on personal and interpersonal skills was found to be significant (the Sig. (2-tailed, *p=*,027). Evaluation of the data from the SHIP-BC survey indicated improvements in scores after participation in the program, supporting the mentees did benefit from the process. From the analysis of the pre-survey scores, most participants score highly and continued to score high in the post-survey. Assessment survey data showed that in all instances, the mentee rated the mentor more competent that the mentor rated themselves. |
| Phelps, 2005, USA | To identify the role formal mentoring programs can play in developing nurse leaders. | Eight mentor and mentee pairs (n=16) in leadership positions at a large community healthcare system in central Virginia. | Action Research. Qualitative and Quantitative measures. Doctoral dissertation. The Leadership Practices Inventory, The Personal Learning Measures and Mentoring Effectivenes Survey. Qualitative data were analyzed using Microsoft word tools to determine word occurrence once the common words were identified. | The three-month formal mentoring program. Consisted of a formal three-hour educational program meeting, and educational and problem-solving sessions. Formal classroom development, website, e-mail, journaling and online communication. | Common words related to the mentoring programs' weaknesses and strengths were time, support, knowledge, experience, and fears or concerns.  During the mentoring process protégés developed and gained skills in communication, conflict management, resources utilization, personal awareness of their leadership style, and recognition strategies.  Personal learning was described as significant by every participant in the Mentoring Leaders Program (n=16). For mentees, both dimensions, job learning (92%) and personal skill development (100%), demonstrated significant positive responses. Personal skills included improvements in communication, listening skills, job performance, sensitivity to others' feelings and attitudes, learning new skills and expanding the way to think about things.  Job learning dimension included improvements in wider understanding and knowledge of the whole organization, it´s politics and other departments functions. |
| Rosser *et al*., 2020, international study | To report on the evaluation of the online Global Leadership Mentoring Community,  a programme designed to build relationships across seven global regions and promote leadership development for emerging nurse leaders. | Eleven mentor- mentee pairs (n=22) who were members of Sigma. Mentees (n=11) were emerging nurse leaders from Australia, Hong Kong, Japan, Lebanon, the Netherlands, Philippines, Taiwan and the USA. | A programme evaluation, outcomes-focused approach. Data were collected using online surveys with open-ended questions and Likert Scale at the outset and end of programme capturing both quantitative and qualitative data. Quantitative data were analyzed using descriptive statistics and for qualitative data, a thematic analysis. | The 18-month mentoring program. Mentors and mentees met monthly using online/virtual technology and quarterly group calls. | There were many successful outcomes of global mentoring relationships including leadership skills, global relationships, ideas sharing and feeling of satisfaction.  There were also challenges including different cultural perspectives, languages and time.  Benefits included broader meetings with other participants as well as specific meetings, mentors' flexibility, clear expectations setting and relationship building.  The mentoring programme increased mentees' involvement in leadership activities and development. It influenced their career decisions and organizational involvement. |
| Roth and Whitehead , 2019, USA | To develop an evidence-based formal mentorship program for NMs in an effort to impact retention rates. | Nurse managers (n = 15) across two hospitals in the Pacific Northwest. | Quasi-experimental, one-group pretest and posttest design. An online survey method using Leadership Practices Inventory -tool and the Nurse Manager Practice Environment scale. Data were analyzed using a paired-samples t test in SPSS Version 24. | The mentorship program was composed of six total sessions spanning over 6 months. In addition, between these sessions' dyads took meetings on online or in person. | There were statistically significant differences between the pre- and postprogram Leadership Practices Inventory scores (p = .013). This demonstrated an increase in leadership competency and transformational leadership behaviors.  There were also statistically significant differences between the pre- and postprogram Nurse Manager Practice Environment Scale–Culture of Generativity subscale (p = .032). This demonstrated an increase in job satisfaction, intent to stay, and transformational leadership behaviors.  14 (93.3%) of the participants reported that peer mentor supports their career development. |
| Waters *et al.,* 2003, Australia | To determine participant expectations of mentoring and outcomes of the pilot programme. | Nurse managers mentors (n=17) and mentees (n=20), in two rural areas of New South Wales and one metropolitan site (the city of Sydney).  Interviews were conducted with 11 of the 37 participants. | A pilot study to determine the feasibility of the programme. Postworkshop telephone interviews semi-structured format. Thematic analysis. Results were analysed using a combination of methods appropriate to the quantitative (descriptive and comparative statistics) and qualitative (thematic analysis) data obtained from the questionnaire and interviews | A pilot programme was developed. The programme included matching and self-selection of mentor and mentee roles and attendance at a full-day Mentoring Workshop. | The pilot mentoring programme was successful in identifying strategies for the development of a tailored and sustainable programme for newly appointed nurse managers. Participants highlighted issues about personal choice and timing, expectations of a structured programme and making greater use of existing technology to support and maintain networks  The mentoring programme provided an opportunity to gain an appreciation of the wider issues, reflect on personal development, network with colleagues and expand leaders' enthusiasm and self-esteem.  Provision of a highly structured and facilitated programme carries high expectations of continued external support. Nurse managers also expressed a desire to choose when, how and whom they would select as mentors. |

**Source**: Authors’ own work
